# A Case of Varicella-Zoster Virus Meningomyelitis in an HIV-1-Infected Patient: Facing the Challenges Related to Its Management and Prognosis

**DOI:** 10.7759/cureus.27652

**Published:** 2022-08-03

**Authors:** Catarina Lameiras, Rita Patrocínio de Jesus, Bárbara Flor-de-Lima, Joana Silva, Patrícia Pacheco

**Affiliations:** 1 Internal Medicine, Hospital Professor Doutor Fernando Fonseca, Amadora, PRT; 2 Infectious Diseases, Hospital Professor Doutor Fernando Fonseca, Amadora, PRT

**Keywords:** hiv infection, meningitis, transverse myelitis, herpes zoster, varicella zoster virus

## Abstract

Varicella-zoster virus (VZV) myelitis is a rare complication of herpes zoster. Diagnosing and treating this entity may be challenging. Clinical outcomes vary and neurological sequelae may be seen despite treatment. We report a case of a 43-year-old woman with human immunodeficiency virus type 1 (HIV-1) infection (CD4 cell count 191 cells/µL - 14%; undetectable viral load) who was started on antiretroviral treatment eight months before. She presented with VZV meningitis and transverse myelitis and concomitant thoracic vesicular rash at the dermatomal level T6. Neurological examination revealed neck stiffness, paraplegia, sensory level below T4, and autonomic dysfunction. Magnetic resonance imaging (MRI) revealed signs of myelitis from C4 to T10 and VZV DNA by polymerase chain reaction (PCR) was positive (20,00,000 cp/mL) in the cerebrospinal fluid (CSF). The patient completed four weeks of intravenous acyclovir and systemic corticosteroids. Repeat lumbar puncture returned negative for VZV PCR and MRI showed spinal cord improvement. However, only partial neurological improvement was observed after six months. Some features of the present case may be associated with an unfavorable outcome, including high VZV viral load in the CSF and rapid progression of neurological deficits to paraplegia and sphincter dysfunction. Moreover, the recovery of CD4+ cells from 4% to 14% after starting antiretroviral treatment might also have contributed to the extension of myelopathy. Further studies are needed to improve the understanding of VZV myelitis course and optimize its treatment.

## Introduction

Varicella-zoster virus (VZV) is a human herpesvirus that causes varicella (chickenpox), becomes latent in cranial-nerve and dorsal-root ganglia, and frequently reactivates causing zoster (shingles). The likelihood of VZV reactivation increases with age and immunosuppression [1]. The most common presentation of VZV reactivation consists of cutaneous zoster. In immunocompromised hosts, VZV reactivation is more likely to cause disseminated disease [1]. VZV may disseminate to the central nervous system (CNS). CNS involvement can lead to meningitis, encephalitis, ventriculitis, polyneuritis, radiculitis, and, less frequently, ischaemic stroke and myelitis [2,3]. VZV myelitis is a rare entity. Inflammatory, demyelinating, and vasculitic mechanisms triggered by VZV reactivation and direct viral invasion of the spinal cord can lead to tissue injury [4,5]. Diagnosing and treating this condition may be challenging, mainly in patients with HIV infection. These patients may present a broad clinical spectrum of neurological complications and some cases can be severe [2,4]. Therefore, physicians must keep a high index of suspicion and be aware of the management of this condition. Full neurological recovery is likely to be observed in up to 75% of cases [6]. However, permanent neurologic damage is possible despite treatment.

## Case presentation

We present the case of a 43-year-old woman who was born in Guinea-Bissau and living in Portugal for the last four years. She had been diagnosed with HIV-1 and hepatitis B co-infection eight months before, with cerebral toxoplasmosis as initial presentation. The nadir CD4+ cell count was 107 cells/uL (4%), with CD4+/CD8+ ratio of 0.06 and HIV viral load of 898,000 copies/mL. She was initiated on tenofovir, emtricitabine, and dolutegravir two weeks after starting treatment with pyrimethamine and clindamycin. Other regular medications included calcium folinate and levetiracetam.

Seven months after starting antiretroviral treatment, she presented to the emergency department with a five-day history of abdominal pain and numbness, nausea, and vomiting. She described muscle weakness of the lower limbs the day prior. On hospital admission, she was pyretic (tympanic temperature of 38.3°C) and presented a left-thoracic vesicular rash compatible with herpes zoster at the dermatomal level T6. Neurological examination revealed severe nuchal rigidity, photophobia, paraplegia with abolished myotatic reflexes of the lower limbs, indifferent right plantar reflex, abolished abdominal cutaneous reflexes, bilateral anesthesia below level T4, apallesthesia below level T9. She also presented with urinary retention and constipation.

Laboratory findings were unremarkable (full blood count, liver enzymes, creatinine, C-reactive protein, and erythrocyte sedimentation rate). CD4+ cell count was 191 cells/µL (14%), with a CD4+/CD8+ ratio of 0.23 and HIV-1 viral load was undetectable (<20 copies/mL). Cranial and spinal computed tomography (CT) showed no acute abnormalities. The cerebrospinal fluid (CSF) revealed pleocytosis (2000 cells/uL, 60% mononuclear), elevated protein count (431 mg/dL), normal glucose levels (53 mg/dL in the CSF, 100 mg/dL in the serum), and normal adenosine deaminase levels. This CSF analysis was in line with a probable infection of the CNS, making the possibility of an autoimmune, parainfectious, or inflammatory cause much less probable.

Assuming the diagnosis of infectious meningitis and myelopathy in an immunocompromised patient, she was empirically started on intravenous ceftriaxone 2g q12h, ampicillin 2g q4h, acyclovir 10 mg/kg q8h (700 mg q8h) and dexamethasone 5 mg q8h. 

**Figure 1:**
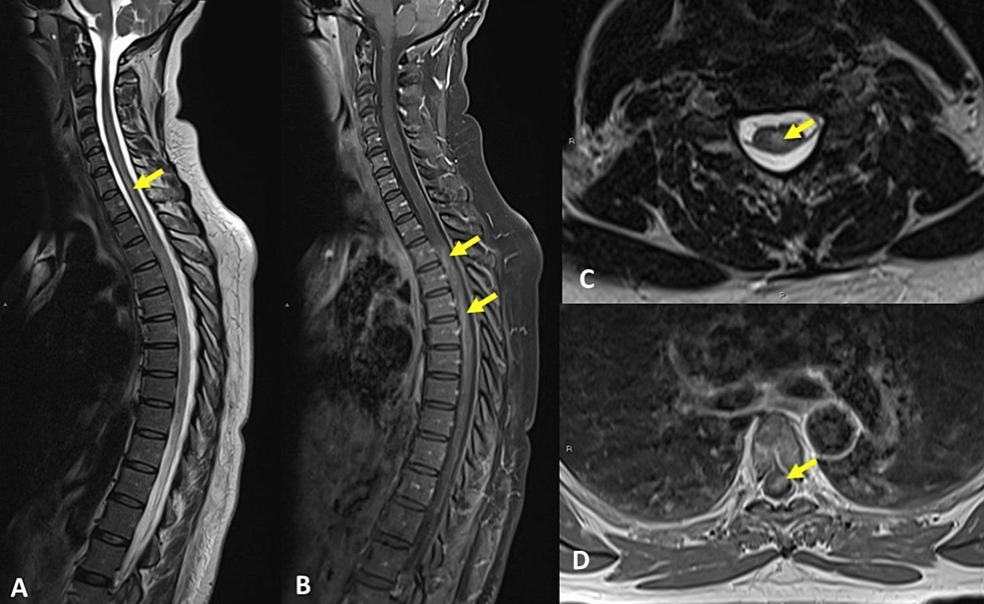
MRI of the spinal cord - (A) sagittal T2-weighted sequence, (B) sagittal contrast-enhanced T1-weighted, (C) axial T2-weighted (C6 level), and (D) axial contrast-enhanced T1-weighted (T5 level). Neuroimages reveal central spinal cord swelling from C4 to T10 and increased T2-weighted signal in C6, C7, and T1 levels (A, C - arrows). Following gadolinium administration, a patchy enhancement of the spinal cord from T2 to T6 was documented, mainly on the left side (B, D - arrows). Pachymeningeal contrast enhancement was also observed (B).

Spinal cord MRI was performed six days later that showed central cord swelling from C4 to T10 and hyperintense signal on T2-weighted sequences (Figure 1, panels A and C). After gadolinium administration, a patchy enhancement from T2 to T6 was documented on T1- weighted sequences, mainly on the left side of the spinal cord (Figure 1, panels B and D).

On the same day, CSF polymerase chain reaction (PCR)returned positive for VZV (20,00,000 cp/mL) and Epstein-Barr virus (EBV) (58,700 cp/mL). CSF remaining PCR results were negative for other herpes viruses (herpes simplex virus-1, herpes simplex virus-2, human herpes virus-6, and cytomegalovirus) and for *Toxoplasma gondii*, *Mycobacterium tuberculosis*, *Chlamydia trachomatis*, and *Neisseria gonorrhoeae*. CSF bacterial capsular antigens, cryptococcal antigen, and bacteriological and mycological cultures were negative. HTLV-1 infection was not investigated since the patient did not present signs or symptoms of upper motor neuron disease. CSF cytological assays showed no evidence of malignancy.

These findings confirmed the diagnosis of herpes zoster meningitis and transverse myelitis. Considering the overall clinical presentation, CSF detection of EBV DNA in the CSF was interpreted as a spurious finding, not directly related to the clinical picture, because EBV DNA may be commonly found in CSF of immunocompromised hosts with other CNS infections or diseases (e.g., CNS lymphoproliferative disorder) [7,8].

Antibiotics were stopped and the patient was treated with intravenous acyclovir 10 mg/kg q8h and oral dexamethasone 4 mg q6h. Since there was only slight clinical improvement, dexamethasone was prolonged for four weeks, then switched to oral prednisolone, tapering off 20 mg every seven days. There were no complications related to steroid treatment. Concomitantly, the patient started rehabilitation. On the 20th day of treatment, MRI showed partial improvement of myelopathy, with reduced extension of the spinal cord lesion. At that time the CSF contained only 4 cells/uL, 87 mg of proteins/dL, and the PCR results of VZV DNA and EBV DNA became negative. However, motor and sensory impairment persisted. At six months of follow-up, she presented partial neurological improvement, with movement of the lower limbs in a horizontal plane, sensory impairment below level T12, and bladder dysfunction.

## Discussion

Herpes zoster myelopathy usually occurs within three weeks of the rash onset. In immunocompromised patients, myelitis is often more insidious and progressive [4]. Neurological symptoms usually begin unilaterally, contiguous with the dermatomal level, and subsequently become bilateral. Motor manifestations are followed by spinothalamic and posterior column sensory abnormalities and bladder or bowel dysfunction [4]. In most cases, the diagnosis of VZV myelitis is presumptive, based on the association of spinal cord dysfunction with the typical zoster eruption. However, myelitis may develop without concomitant rash, mainly in immunocompromised patients [4]. Atypical presentations of VZV myelitis in immunocompromised patients make clinical suspicion even more challenging.

MRI findings are usually non-specific. Most commonly, spinal cord enlargement and focal or diffuse intramedullary T2-hyperintensity are found. Patchy contrast enhancement occurs in some cases [9,10]. The diagnosis can be supported by the detection of VZV DNA by PCR or antibodies in the CSF. Intrathecal detection of specific antibodies to the virus is delayed and can be difficult in immunocompromised patients [3]. Detection of VZV DNA in the CSF has been shown to be the most specific and rapid tool for early diagnosis [4]. However, interpretation of its presence in the CSF must be cautious when it is also present in the serum, especially in immunocompromised patients [9]. Moreover, false-negative VZV PCR can occur, due to the timing of sampling. VZV DNA by PCR is detectable during the early days after the onset of rash and rapidly declines within 10 days, while anti-VZV IgG antibody levels increase from the first week and persist during the clinical course [4,5]. Therefore, both PCR and antibody analysis are recommended. In the appropriate clinical setting, the diagnosis should not be ruled out even if no VZV DNA or specific IgG is detected. For these reasons, the diagnosis of VZV myelitis is best made by considering the appropriate clinical setting, suggestive laboratory evidence, and alternative diagnoses.

There is no established treatment regimen for VZV myelitis since no clinical trials have been done to determine its efficacy. The pathological finding of active viral infection as the basis of cord injury is an indication for antiviral therapy and high doses of intravenous acyclovir are recommended [4,6]. The use of corticosteroids is less clear, but the evidence of vascular inflammation in tissue specimens from patients with CNS sequelae of VZV infection is a rationale for concomitant corticosteroid therapy [4-6].

The spectrum of clinical outcomes in VZV myelitis ranges from spontaneous recovery to ascending progression and death. Neurological sequelae may be seen despite treatment. Immunosuppression may influence the severity of myelitis and be associated with less favorable clinical course and long-term prognosis, but more evidence is needed [11,12]. Rapid progression of neurological deficits, paraplegia and flaccidity, and bladder dysfunction are usually associated with an unfavorable outcome [4,12].

In our case, meningitis and transverse myelitis related to herpes zoster were diagnosed based on typical neurological manifestations with simultaneous zoster rash, MRI spinal cord findings, and CSF VZV PCR detection. Early treatment with high-dose intravenous acyclovir and steroids was implemented. Although follow-up studies revealed radiological and microbiological improvement, the patient still presented neurological sequelae. 

Even though there is no consensus regarding VZV myelitis treatment, some of the cases reported in the literature were initially treated with methylprednisolone 1 g/day [13-15]. Our patient was started on a lower steroid dosage, due to the already ongoing symptoms for some days at the time of the definitive diagnosis, which may have influenced the less favorable outcome. However, there is no evidence of the efficacy of steroids in the treatment of VZV myelitis and some cases recover spontaneously or without the use of steroids [16,17]. In the cases described in the literature, treatment decisions, such as steroid dosage and treatment duration, were made on a case-by-case basis, with few standards available to predict treatment outcomes (Table 1).

**Table 1:** Summary of some published cases of VZV myelitis with different treatment strategies. AIDS: acquired immunodeficiency syndrome; IRIS: immune reconstitution inflammatory syndrome; IV: intravenous; VZV: varicella-zoster virus

Case report	HIV infection status	Treatment	Outcome
Yýlmaz et al. (case 1) [14]	Negative	IV methylprednisolone 1 mg/kg/ day for two weeks, tapered by 4 mg each week until 10 mg/day	Partial recovery
Yýlmaz et al. (case 2) [14]	Negative	Oral methylprednisolone 20 mg/day for three weeks, tapered by 4 mg every week until 4 mg/day	Partial recovery
Abbas et al. [15]	Negative	IV acyclovir 10 mg/kg every 8 hours for 21 days plus IV methylprednisolone 1 g/day for three days followed by oral tapering	No improvement
Toledano et al. [16]	Negative	IV acyclovir 10 mg/kg every 8 hours for 14 days	Complete recovery
Lionnet et al. (case 1) [17]	HIV-1 (AIDS)	IV acyclovir 30 mg/kg/day for 35 days, then switched to oral acyclovir 1 g/day (duration not specified)	Partial recovery
Lionnet et al. (case 2) [17]	HIV-1 (AIDS)	IV acyclovir 30 mg/kg/day for 21 days, then switched to oral acyclovir 1 g/day (duration not specified)	Complete recovery
Clark et al. [18]	HIV-1 (IRIS?)	Valacyclovir (duration and dose not specified) plus IV methylprednisolone 1 g/day for three days	Partial recovery
Newsome et al. [19]	HIV-1 (IRIS?)	IV methylprednisolone 1 g/day for 5 days. Worsening 48 hours after steroids were stopped, therefore IV methylprednisolone was reinstituted for three days and then transitioned to prednisone 1 mg/kg/day (duration not specified)	Partial recovery

The short latency between herpes zoster and neurologic symptoms of the present case, as well as the progression to limb paraplegia with sphincter dysfunction, may be associated with the less favorable course observed. Our patient also presented a high VZV viral load in the CSF, which has also been correlated with the severity of neurologic disease [20].

Moreover, the recovery of CD4+ cells from 4% to 14% after starting antiretroviral treatment might also have contributed to the extension of myelopathy since immune reconstitution inflammatory syndrome may possibly contribute to the severity of CNS involvement [10]. Immune reconstitution inflammatory syndrome in HIV-infected patients after initiating antiretroviral therapy is not a completely well-understood phenomenon. Potential mechanisms include a partial recovery of the immune system or exuberant host immunological responses to antigenic stimuli. VZV is one of the infectious agents commonly associated with this syndrome [10]. VZV myelitis with possible contribution to immune reconstitution inflammatory syndrome is seldom described in the literature [10,18,19]. The reported cases present some similarities to our case, including similar MRI findings and partial/permanent neurological disability. Considering the possible contribution of robust inflammatory response to our case of myelitis, steroid treatment duration was prolonged. Since prolonged use of steroids in immunocompromised patients carry additional risks, their use should be cautious and carefully monitored.

## Conclusions

This case illustrates the adversities related to the management and treatment of VZV myelitis in immunocompromised patients with HIV infection. Even with institution of treatment, it may be associated with poor prognosis. VZV myelitis may be more severe in immunocompromised patients. In our case, the rapid development of neurological deficits, progression to paraplegia and autonomic dysfunction, the high VZV viral load in the CSF, and the possible association with immune reconstitution inflammatory syndrome might have contributed to the severity of the CNS involvement and less favorable outcome. Further investigation is needed to improve the understanding of VZV myelitis course and mechanisms and the factors influencing its outcomes, to allow optimization of well-established treatment strategies.
